# The impact of iron-biofortified bean adoption on bean productivity, consumption, purchases and sales

**DOI:** 10.1016/j.worlddev.2020.105260

**Published:** 2021-03

**Authors:** Kate Vaiknoras, Catherine Larochelle

**Affiliations:** a USDA Economic Research Service, 805 Pennsylvania Ave, Kansas City, MO 64105, United States; b Virginia Tech, Department of Agricultural and Applied Economics, 250 Drillfield Drive, 315 Hutcheson Hall, Blacksburg, VA 24061, United States

**Keywords:** Iron-biofortified beans, Africa, Rwanda, Impact, Instrumental variables, Control function approach

## Abstract

Staple food crops tend to be low in micronutrients; therefore, individuals whose diets rely heavily on them can suffer from micronutrient deficiency. Biofortification addresses this issue through the breeding of staple crops that are micronutrient-dense and high yielding. One such crop is iron-biofortified beans. Ten iron-biofortified bean varieties were released between 2010 and 2012 in Rwanda, a country with high rates of bean production and consumption, to address iron deficiency. This study evaluates the effect of the most widely adopted of these varieties, RWR2245, on household yield, land cultivated under beans, bean consumption, purchases, and sales. Because the adoption decision could be endogenous, we use a control function approach to quantify the impacts of adoption. RWR2245 provides a yield gain of 20%-49% over traditional bush bean varieties. In our preferred model specification, we find that over a 12-month period, growing RWR2245 for at least one out of two annual growing seasons increases the length of time beans are consumed from own production by 0.64 months (19–20 days), reduces the length of time beans are purchased for consumption by 0.73 months (22–23 days), and increases the probability of selling beans by 12%. Adoption can thus improve household nutrition via two channels: primarily by increasing iron intake via substituting biofortified harvested beans for less nutrient-dense beans from the market, and additionally by increasing household income that can be spent on nutritious foods through the reduction in bean purchases and increased likelihood of selling beans. Moreover, the sale of iron-biofortified beans implies the availability of iron-dense food in markets, also benefiting households that purchase beans. These findings are promising for the continued adoption of iron-biofortified beans in Rwanda and elsewhere and provide evidence that biofortified crops are an effective investment for nutrition, food security, and poverty reduction.

## Highlights

• We estimate the impact of adoption of RWR2245, a popular iron-biofortified bean released in Rwanda.
• Adoption provides a yield gain of 20%-49% over that of local bush bean varieties.
• Adoption increases household consumption of beans from own production and reduces bean purchases.
• Adoption increases the probability that households sell beans by 12%.
• RWR2245 increases iron intake among adopting households and availability of iron-rich food sources in the market.

## 1 Introduction

Throughout the world, families subsist on diets high in staple crops and low in diversity due to a lack of income to purchase or produce more nutritious foods. Even when calorie intake is sufficient, staple foods are often low in vitamins and minerals. This can lead to micronutrient deficiency, which affects over two billion people around the world. Iron deficiency, which causes anemia, fatigue, increased risk of infection, and pregnancy complications, is one of the most common micronutrient deficiencies globally (World Health Organization, 2018). Iron deficiency in young children can impair cognitive and physical development, resulting in lower educational achievement and productivity, which in turn is associated with a lifetime of reduced earning opportunities, perpetuating poverty (Alderman et al., 2006).

In the past few decades, crop breeding has greatly increased yields of staple crops. More recently, scientists have turned to crop breeding to develop a new intervention to address the nutritional needs of families whose diets are high in staple crops: biofortification, a process through which staple food crops are bred to be both high yielding and high in micronutrients. Over 290 biofortified varieties across 12 crops have been released or are being tested in over 60 countries (Meyer, 2018). One such biofortified crop is iron-biofortified beans, designed to combat iron deficiency. Randomized control trials have established that consumption of iron-biofortified crops reduces iron deficiency and improves functional outcomes (De Moura et al., 2014, Finkelstein et al., 2017, Luna et al., 2015, Murray-Kolb et al., 2017). Iron-deficient women in Rwanda who consumed iron-biofortified bean varieties instead of non-biofortified varieties for 18 weeks experienced improved memory and ability to pay attention (Murray-Kolb et al., 2017) and a reduction in time spent in sedentary activities (Luna et al., 2015).

Biofortification is most cost-effective when micronutrient deficiencies of the poor can be addressed by foods they commonly produce and consume (Meenakshi et al., 2007). For this reason, Rwanda was selected as the first country for the release of iron-biofortified beans. Rwanda is a small country that has a high population density and heavy reliance on agriculture, and where beans make up 32% of caloric and 64% of protein intake for the average household (Mulambu et al., 2017). Rwanda has two cropping seasons per year, season A, which runs from September to January, and season B, which runs from February to August. Most farmers produce beans in both seasons (Asare-Marfo et al., 2016a).

In collaboration with the International Center for Tropical Agriculture and HarvestPlus, the Rwanda Agricultural Board released four iron-biofortified bean varieties in 2010 and six more in 2012. These varieties have approximately twice the iron content of non-biofortified varieties, are adapted to local conditions, high-yielding, and resistant to common pests and diseases. They have a wide range of colors, sizes, and agronomic and consumption characteristics to appeal to bean producers and consumers throughout the country. Two of the released varieties are bush bean varieties with potential yields of 2–2.5 t/ha (Katsvairo, 2014). The remaining eight varieties are climbing bean varieties that can yield between 3 and 4.5 t/ha but require additional inputs including stakes and a higher rate of fertilizer application to achieve these high yield potentials (Katsvairo, 2014). Climbing varieties tend to be most popular in the North of the country which is characterized by higher elevations, greater land scarcity, more rainfall, and cooler temperatures.

Since 2012, HarvestPlus and its partners have promoted and distributed iron-biofortified bean planting material to small landholders in Rwanda through several delivery approaches, resulting in fast and sustained adoption of the varieties (Vaiknoras et al., 2019). The approach that has reached the greatest number of farmers is direct marketing which consists of selling small packets (ranging from 200 to 500 g) of iron-biofortified bean seed to farming households in local markets. By the end of 2015, over a quarter of a million households accessed iron-biofortified seed through direct marketing (Mulambu et al., 2017). Other approaches used to distribute iron-biofortified seed include seed swap and payback. Under seed swap, farmers and cooperatives receive iron-biofortified seeds in exchange for seeds of other bean varieties while in the payback system a portion of harvested iron-biofortified grain is given back to HarvestPlus and its collaborators. Agrodealers also sell iron-biofortified seeds in shops. Finally, iron-biofortified planting material spreads through informal dissemination via social networks (Vaiknoras et al., 2019).

As a result of both formal delivery and informal dissemination, iron-biofortified bean varieties are widely adopted in Rwanda; approximately 28% of rural households grew an iron-biofortified bean variety for at least one season between 2012 and 2015 (Asare-Marfo et al., 2016b). The most popular iron-biofortified bean variety in Rwanda is RWR2245, a bush variety planted by over half of households that have grown an iron-biofortified bean variety. RWR2245 has also been the most widely disseminated variety; in most seasons, it represents over 70% of iron-biofortified bean planting material delivered via formal approaches. The other iron-biofortified bush bean variety, RWR2154, has been planted by about 2% of households that have grown an iron-biofortified bean variety; the remaining iron-biofortified bean adopters have grown one or more of the eight climbing varieties (Asare-Marfo et al., 2016b).

While the nutritional and health benefits of consuming iron-biofortified crops are well-established (De Moura et al., 2014), the literature has not yet widely explored the impacts of biofortified crop adoption on earlier links along the impact pathway such as on yield, consumption, and sales of the targeted crop. De Brauw et al. (2018) examine the adoption and impact of another biofortified crop, orange-fleshed sweet potato (OSFP), which is biofortified with vitamin A. They find that the HarvestPlus Reaching End Users (REU) program, which distributed OSFP planting material and provided nutrition education to participants in Uganda and Mozambique, has been successful in increasing adoption of OSFP, nutritional knowledge, and vitamin-A intake of children. They explore impact pathways of REU and conclude that the improved nutritional intake stems more from its effect on adoption rather than through nutrition education. Our paper differs from de Brauw et al. (2018) in two significant ways; first, we examine biofortified beans rather than sweet potatoes and second, we explore a different set of impact pathways.

Extensive research has also been conducted on beans in Rwanda. Larochelle et al. (2015) document the impact of improved bean varieties on yield and food security in Rwanda and Uganda while Katungi et al. (2018) examine the association between climbing bean cultivation and household wellbeing in Rwanda. The first study differentiates improved and local bean varieties and the second one compares climbing and bush bean growing households. Both studies were conducted before the start of the intensive dissemination efforts of iron-biofortified bean varieties.

Vaiknoras et al. (2019) establish that adoption of iron-biofortified beans in Rwanda is widespread and has grown over time, but the authors do not estimate the impacts of adoption on household outcomes. Two published reports provide descriptive analyses of adoption of iron-biofortified beans in Rwanda (Asare-Marfo et al., 2016, Asare-Marfo et al., 2016). One of these reports also provides descriptive statistics of yield differentials between iron-biofortified and local bean varieties but the analysis does not control for covariates that can influence yield and does not address selection bias (Asare-Marfo et al., 2016b). Nsengiyumva et al. (2017) use propensity score matching (PSM) and find that adoption of RWR2245 in Nyagatare district, Eastern Province of Rwanda, increases yield by 367–810 kg/ha while Funes et al. (2019), also using PSM, estimate that adoption of iron-biofortified varieties increases average household yield by 22–23% and potential agricultural income by 24–25%. However, more research is needed that measures the impact of adoption on a wider range of indicators and that takes into account the endogeneity of the adoption decision.

Our paper fills this gap by using nationally representative rural household data to quantify the impacts of RWR2245 adoption on first- and higher-order household outcomes. The specific objectives of this study are to estimate the impact on yield, which is the first-order effect of adoption, and to quantify the impacts on the following higher-order outcomes: changes in land area under bean cultivation, bean consumption from own production and purchases, bean sales, and the likelihood of being a net seller of beans. We use a control function approach (CFA) which makes use of instrumental variables (IVs) constructed from rollout data of promotion of iron-biofortified planting material in local markets and informal dissemination to identify the causal effects of adoption. Direct marketing is strongly correlated with adoption but should not be correlated with our outcomes of interest, as the rollout was not related to bean production, consumption, or marketing characteristics of local communities. Informal dissemination, captured using the previous-season village adoption rate for RWR2245, increases the likelihood of adoption by increasing the quantity of planting material within one’s social network, but should otherwise be exogenous to the outcomes of interest.

Findings from this study will inform the development of future programs promoting biofortification, better position decision-makers to justify funding for biofortification, and potentially guide the allocation of resources towards programs with the greatest impacts. Rigorously quantifying the yield gain from adoption enhances the identification of the higher-order outcomes that stem from higher productivity. It also informs development practitioners, researchers, and policymakers of the yield achieved under farmer specific conditions. The distributional benefits of biofortified crops depend on their harvest usage. If adopters consume most of their biofortified harvest, we expect that benefits will mainly accrue to adopters themselves, indicating that policymakers should target the promotion of the crops to households with high nutritional needs, such as those with young children. By contrast, if adopters sell most of their biofortified harvests then the nutritional benefits will spread to households who purchase grain from adopters; thus, policymakers should target households with large landholdings and bean production.

The next section describes our data and is followed by a discussion of our conceptual and empirical framework, including estimation techniques, choice of variables and robustness checks. We then present descriptive analysis and econometric findings and conclude with policy implications that emerge from our findings.

## 2 Data source

This study uses nationally representative data of bean producers in Rwanda collected in two stages. The first stage was a listing exercise that occurred in May and June 2015, which corresponds to mid-season 2015B. One hundred twenty villages were randomly selected and all households in the selected villages were interviewed, totaling 19,575 households, regarding iron-biofortified bean adoption histories from seasons 2010A to 2015B. To assist respondents in accurately identifying iron-biofortified beans, enumerators showed them a seed sample of the ten varieties, one at the time. They asked respondents if they had ever seen or heard of the variety, if they had ever grown it and if so, which cropping season they had first adopted the variety, and then whether the respondents had grown the variety in each subsequent season. Further questions were asked to verify that the household correctly identified the variety, including the color and bean type (i.e. bush or climbing) of the variety.

Twelve households in each village (six iron-biofortified bean adopters and six non-adopters, when possible)^1^ were re-interviewed for the main household survey, which took place in September 2015, after season 2015B harvest. The respondent was the household member who was most knowledgeable about bean cultivation in the household. Enumerators collected the following information for all bean varieties grown during season 2015B: whether it was an iron-biofortified, improved (but not biofortified), or local/traditional variety, the quantity of seed planted, and the quantity of grain harvested. For each bean plot cultivated that season, the respondent reported the size, distance from their home, input usage, and identified the household member responsible for making cropping decisions for the plot. Respondents reported the months, out of the last 12 months, that the household consumed beans from own production, the months that they purchased beans in the market for home consumption, and the average quantities consumed monthly when beans were sourced from the farm and the market. They also reported the total quantity of beans sold in the last 12 months. Finally, the survey elicited information on household demographics, housing characteristics, and asset ownership.

We also use data from a community survey and HarvestPlus delivery records for direct marketing. The community survey was implemented at the same time as the main household survey. Key informants were interviewed, including the village leader, regarding village characteristics such as access to extension and the presence of iron-biofortification delivery activities. We use HarvestPlus direct marketing delivery records to compute the number of locations where direct marketing occurred in each sector (an administrative unit between village and district) for seasons 2015A and 2015B.

Because we want to measure the impacts of RWR2245 adoption, which is a bush bean variety, we restrict our sample to households that grew at least one bush bean variety^2^ in season 2015B.^3^ The characteristics of households that grew only climbing beans are expected to vary significantly from those of bush bean growers as climbing beans are more popular in the North of the country where land scarcity and poverty are more prevalent than in the remainder of the country. Moreover, management practices and yield potential differ between bush and climbing beans. Therefore, the analyses in this paper are based on 815 bush bean growing households who grew an average of 1.37 bush bean varieties in 2015B, for a total of 1112 variety-level observations.^4^

## 3 Conceptual framework and empirical estimations

### 3.1 Conceptual framework

**Fig. 1:**
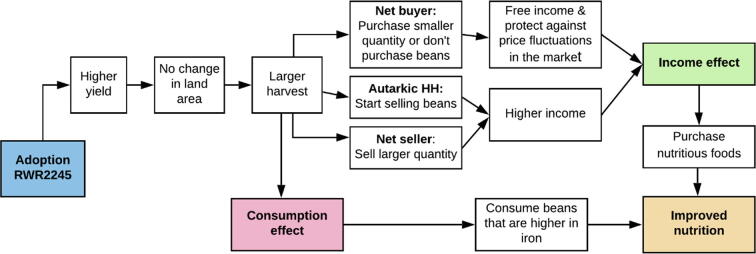
Impact pathway of RWR2245 adoption.

There are many impact pathways through which agricultural interventions can improve nutrition (Webb, 2013). Two commonly identified pathways are improvements in nutrition through: (1) increased consumption of own-produced food, particularly if that food is highly nutritious such as biofortified crops; and (2) increased household incomes via sales of own-produced food, which can be spent on healthy foods such as animal-source proteins or other health-related goods (Pandey et al., 2016, Webb, 2013). In our impact pathway framework, adapted from Pandey et al. (2016), we hypothesize that adoption of RWR2245 improves household nutrition through these consumption and income effects which stem from an increase in harvested beans that arises when yield increases and land under bean cultivation remains the same (two assumptions which we test) (Fig. 1). Among RWR2245 adopters, bean consumption from own production will improve nutrition all else held equal, as biofortified beans have higher iron density than other varieties that the household would have otherwise grown or purchased in the market. This means that iron intake will be higher than it would have been in the absence of adoption (pathway 1: consumption effect, which is represented by the pink box in Fig. 1). We hypothesize that the income effect arises from a more abundant harvest which, assuming no change in production costs^5^ due to adoption, will increase household income (pathway 2 – income effect, which is represented by the green box in Fig. 1).

The source of the income effect varies with the household position in the bean market. For net buyers, the increased production will reduce the quantity of beans purchased in the market to meet household consumption needs, freeing income to spend on other goods and possibly leading to autarky. Greater food self-sufficiency can protect households against price fluctuations in the market and is often used as a proxy for, or a component of, food security (Noromiarilanto et al., 2016, Rufino et al., 2013, Traore et al., 2017). The more abundant harvest may induce autarkic households,^6^ i.e. those that neither purchase nor sell beans, to become net sellers of beans, and increase the quantity sold among current net sellers. Increased household incomes due to a reduction in the quantity of beans purchased or higher bean sales will lead to improved nutrition if the income gain is used to purchase nutritious food. We will examine whether RWR2245 adoption leads to changes in the quantity of beans consumed from own production and the market, quantity of beans sold, and household likelihood of being a net seller of beans. However, we do not have the data to explicitly measure the impact of RWR2245 adoption on household income or changes in food consumption expenditures.

### 3.2 Empirical framework

We estimate the impacts of RWR2245 adoption on yield, a first-order effect of adoption, of bush bean variety *j* grown by household *i* using the following equation:

(1) $$M_{\mathrm{ij}}=\beta_{0}+\beta_{1}T_{\mathrm{ij}}+\beta_{2}I_{ij}+\beta_{3}H_{i}+e_{\mathrm{ij}}$$

The dependent variable in Eq. (1) is the multiplication ratio $M_{\mathrm{ij}}$, which we use as a proxy for yield. The multiplication ratio is the quantity of bush bean grain harvested by household *i* for variety *j* divided by the quantity of seeds planted for that variety and household. We use the multiplication ratio instead of yield because we expect the former to provide a more accurate measure of bean productivity. This is because estimates of plot areas are more prone to measurement errors than quantity planted and harvested, especially in Rwanda where plots are often of irregular shapes and intercropped. In Rwanda, households grow both local varieties and high-yielding improved varieties. We expect RWR2245 to provide a higher multiplication ratio than local bush varieties, but a similar one to other improved varieties. Thus, $T_{\mathrm{ij}}$ is a categorical treatment variable equal to 0 if variety *j* is a local bush variety, 1 if it is an improved bush variety other than RWR2245, and 2 if variety *j* is RWR2245. As a result, the comparison group in our estimation is local bush varieties.

We control for agricultural inputs used in the cultivation of variety *j* and plot characteristics on which variety *j* is grown; these covariates enter the vector $I_{ij}$ in Eq. (1). Covariates capturing input use are whether organic fertilizer was applied, whether chemical fertilizer was applied, and the source of planting material, i.e. whether the planting material is from recycled seed vs. sources including local markets, farmer groups, or HarvestPlus delivery approaches. The term recycled seed refers to grain harvested by a farmer during the past season and saved to be used as seed for this planting season. Plot characteristics include the slope of the plot (flat, gentle, moderate, or steep), whether the plot was intercropped, and the distance from the household to the plot (in minutes walking). The vector $I_{ij}$ also includes the sex, literacy, and years of experience growing beans of the household member who makes planting decisions for the plot on which variety *j* was grown since the decision-maker about bean production activities varies across plots for household *i*. Household-level variables that could influence productivity and are potentially correlated with adoption of RWR2245 also enter the regression and are represented by the vector $H_{i}$. These household-level covariates that do not vary within a household are dwelling elevation, the number of adults in the household as a proxy for farm labor availability, the number of pieces of agricultural equipment owned, and the percentage of households in the village who obtain advice from agricultural extension agents, which captures access to extension and is measured at the village level to avoid endogeneity. Last, we also include province fixed effects to control for differences in agricultural potential, infrastructure, etc. between provinces.

We estimate the impact of RWR2245 adoption on the higher-order outcomes using the following equation:

(2) $$O_{i}=f\left(A_{i},X_{i,}\gamma \right)+e_{i}$$

where the dependent variable $O_{i}$ is the outcome of interest for household *i,*$A_{i}$ is the treatment variable, $X_{i}$ is a vector of exogenous variables, and γ is a vector of coefficients. The vector $O_{i}$ includes eight dependent variables representing land under bean production, bean consumption from own production, bean consumption from purchases, and bean sales. We use the quantity of seed planted in kg in season 2015B as a proxy for land area under bean production since it is less prone to measurement errors than household estimates of land sizes.^7^ We measure bean consumption from *own production* using two variables: i) the number of months in the past 12 months prior to data collection that household *i* consumed beans from own production and ii) the average quantity of beans consumed monthly during the months beans were sourced from own production, measured in kg per adult male equivalent.^8^ Adult male equivalent is used to approximate the food requirements of the household based on the proportional energy requirements of household members of different gender and age compared to an adult male, which is the standard reference (Dary & Hainsworth, 2008). Taking into account the food requirements of different household members provides a more accurate indicator of household food consumption adequacy than a simpler per-capita measure. Likewise, bean consumption *from purchases* are captured using: i) the number of months in the 12 months prior to data collection that the household purchased beans for consumption and ii) the average quantity of beans purchased monthly, in kg per adult equivalent, when beans consumed were from the market. Indicators representing bean sales are: i) whether household *i* sells beans, ii) the quantity of beans sold in the past 12 months, in kg, and iii) whether household *i* is a net seller of beans, which is determined by comparing total bean purchases with total bean sales in the past 12 months. The indicator for net seller is equal to 1 if total sales is equal to or exceeds total purchases, and zero for net buyers and autarkic households.

The treatment variable $A_{i}$ in Eq. (2) is a binary indicator for RWR2245 adoption and varies by dependent variable. When the dependent variable is land area devoted to bean production in season 2015B, $A_{i}$ is equal to one if household *i* grew RWR2245 in season 2015B and zero otherwise. For the remaining outcomes of interest, $A_{i}$ is equal to one if household *i* grew RWR2245 in season 2015A only, in season 2015B only, or in both seasons 2015A and 2015B and zero otherwise. This approach is used for consistency between the treatment variable and the outcomes of interest. The outcomes of interest related to bean consumption and sales cover the 12 months prior to data collection, which spanned over seasons 2015A and 2015B. Thus, the treatment variable $A_{i}$ takes into account the adoption decision in both seasons. We hypothesize that adoption, through higher production, will increase bean consumption from own production while reducing consumption of purchased beans. This may occur through increasing (decreasing) the number of months households consume beans from own production (purchases) and/o and/or increasing (decreasing) the average monthly quantity the household consumes from own production (purchases). Last, we expect adoption to increase the probability that a household sells beans, the quantity of beans sold, and the probability of being a net seller of beans due to RWR2245′s higher yield compared to local varieties.

We control for household-level covariates, which are represented by the vector $X_{i}$ in Eq. (2), that could affect the higher-order outcomes of interest $O_{i}$. These variables include distance to the nearest city of 50,000 people (in km) and population density (in inhabitants/square km) as proxies for proximity to markets, elevation (in meters), the number of household members, the sex, literacy, and age of the respondent, land area cultivated in 2015B (in hectares), a wealth index^9^ created using polychoric principal components analysis, the number of pieces of agricultural equipment owned, livestock ownership measured in tropical livestock units,^10^ and access to extension by households in the village. Moreover, we control for whether household *i* grew climbing beans in 2015B as climbing beans have higher yields on average than bush beans and could thus impact bean production, consumption, and sales (Katungi et al, 2018). Finally, we include province fixed effects as in the yield regression.

There may be household or variety-level factors that are correlated with adoption and the outcomes of interest in Eqs. (1), (2) that are not controlled for, such as unobserved farmer ability or access to resources. As a result, the adoption variables could be correlated with the error terms, *e_ij_* and *e_i_*, causing the treatment effect estimates to be biased. To address this problem, we use a CFA.

### 3.3 Estimation strategies

We use a CFA to address the endogeneity of adoption as this estimator is more efficient than two-stage least squares (2SLS) when the casual effect model is non-linear (Imbens & Wooldridge, 2007). A CFA is a two-stage procedure that tests and controls for endogeneity. In the first stage, the treatment variable is regressed on a set of explanatory variables and IVs. The generalized residuals from this regression are collected. In the second stage, the outcome variable is regressed on the treatment variable, other explanatory variables, and the generalized residuals from the first-stage regression. A *t*-test of the coefficient on the generalized residuals tests the null hypothesis that the treatment is exogenous (Wooldridge, 2014). A CFA can thus provide additional evidence beyond the Hausman test as to whether adoption is endogenous to our outcomes of interest and if so, allow us to estimate causal effects of adoption by controlling for its endogeneity. When adoption is a categorical variable, $\left(T_{\mathrm{ij}}\right)$, we estimate an ordered Probit model in the first stage. When adoption is measured using a binary indicator (*A_i_),* the first stage regression is estimated using a Probit model.

The second stage estimation methods vary depending on the nature of the dependent outcome variable. When the dependent variable is continuous, as it is for the multiplication ratio, quantity of bean seeds planted, and average quantity of beans consumed monthly when beans are sourced from own production and the market, we use ordinary least squares (OLS) in the second stage. We assume a log-linear function because the distribution of these outcomes is highly skewed to the right.

For quantity sold, we estimate a double hurdle model, also called Craggit (Burke, 2009), in the second stage because bean sales are censored, with a large number of observations at zero. Since factors that affect the decision to sell beans may differ from those that influence quantity sold, it is appropriate to model these two processes separately. The double hurdle model consists of estimating a probit model in the first stage and a truncated normal regression in the second stage. Combining the first and second stage estimates, we calculate the average partial effect of adoption on the quantity of beans sold (Burke, 2009). Unlike the Tobit model, the double hurdle model does not require the explanatory variables to have the same effect on the decision to sell beans and the quantity sold. We test the double hurdle model against the Tobit model to determine which model fits the data best.

When the dependent variable is the number of months beans were consumed from own production, we estimate a Poisson model. For the number of months beans were purchased, we estimate a zero-inflated Poisson model because some households did not purchase beans over the past 12 months. Similar to the double hurdle model, the zero-inflated Poisson model allows the decision to purchase beans to be modeled separately from the number of months beans are purchased. We test whether the zero-inflated Poisson model or the standard Poisson model fits the data better using a Vuong test. Finally, for the decision to sell beans and whether the household is a net seller, we estimate Probit models as these are binary outcomes.^11^

Standard errors are robust to heteroskedasticity and clustered at the household level when the dependent variable is the multiplication ratio since several households cultivate more than one bush bean variety, resulting in more than one observation per household. For the other models, standard errors are also robust to heteroskedasticity but clustered at the village level. Because iron-biofortified bean adopters were oversampled during data collection, we use sampling weights in all descriptive and econometric analyses to obtain results that are nationally representative of bean producers in Rwanda.

### 3.4 Choice of instrumental variables

CFA requires the inclusion of IVs in the first stage regression. A relevant and valid IV is one that is correlated with the treatment variable and uncorrelated with the outcome of interest (Wooldridge, 2010). We use two IVs. The first one is the number of locations where HarvestPlus sold iron-biofortified bean seeds in local markets in a household’s sector during seasons 2015A and 2015B. Direct marketing is highly correlated with the adoption of biofortified bean varieties (Vaiknoras et al., 2019). The locations of the direct markets selected for the promotion and sale of iron-biofortified seed were chosen independently of bean productivity, consumption, purchases, and sales of local households. Direct marketing is therefore exogenous to our outcomes of interest.

Our second IV is the village adoption rate for RWR2245 in the season prior to the one in which the outcome variables are measured. This is a proxy for the availability of RWR2245 planting material within a household’s social network. The previous season village adoption rate strongly correlates with current season adoption (Vaiknoras et al., 2019) and is exogenous to an individual household’s yields, consumption, purchases, and sales in the current season as it is based on other farmers’ previous adoption decisions. Like the treatment variable, the seasons considered when measuring village adoption rate vary by dependent variables. When the dependent variables are yield and land under bean production in 2015B, we use the village adoption rate for RWR2245 in season 2015A because in these regressions adoption is defined as growing RWR2245 in 2015B. For the remaining dependent variables, the IV is the village adoption rate for RWR2245 in season 2014B because adoption is defined over 2015A and 2015B. We test the validity of the IVs using a series of diagnostic tests for 2SLS regressions (Baum et al., 2002).

### 3.5 Robustness checks

To test the validity of our results, we implement several robustness checks. We use PSM to evaluate the treatment effect of adoption on first- and higher-order outcomes. For the multiplication ratio, we first estimate a logit model to evaluate the propensity score, or the probability that variety *j* will be RWR2245, based on observable characteristics in ***I_ij_*** and ***H_i_***_._ Then propensity scores are used to match multiplication ratios for RWR2245 and local bush bean varieties that are “similar,” i.e. have propensity scores that are close to one another. We only compare RWR2245 to local bush varieties because we are interested in the yield gain of RWR2245 over local varieties rather than other improved varieties. For the higher-order outcomes, we compute the propensity score of a household being an RWR2245 adopter and compare the outcomes of RWR2245 adopters and non-adopters that have similar propensity scores. The final step is to estimate whether these similar varieties (households) have systematically different outcome values (Austin, 2011).

There are several ways to match control and treatment observations when doing PSM. As a sensitivity check, we use two common algorithms for matching: radius matching, and kernel matching (Caliendo & Kopeinig, 2005). We set a caliper requirement that matches must have propensity scores that do not differ by more than 0.01. We use the observations in the common support, which is the area where propensity scores between the treatment and comparison groups overlap, to improve matching (Caliendo & Kopeinig, 2005).

PSM only explicitly controls for overt bias, meaning that if hidden bias (based on unobserved characteristics) is present, results will be biased. An advantage of PSM is that it requires fewer functional form assumptions about the relationship between the outcomes of interest and explanatory variables, which is why we use it to assess the robustness of our results. To assess how well PSM controls for overt bias, the balance of matched observations must be examined as this provides evidence of how comparable the treatment and control groups are post-matching. One way to do this is to compute the standardized bias, which quantifies the balance. Harder et al. (2010) consider a covariate to be balanced if its standardized bias is less than 0.25, although they recommend using a stricter cut-off of 0.10 when possible, particularly for covariates that are highly correlated with the outcome variable to ensure that the treatment and control groups are comparable and matching estimates are unbiased.^12^

For the multiplication ratio regression, we also estimate a fixed effects (FE) model using a sub-sample of households who grew RWR2245 and at least one other bush bean variety in 2015B. Reducing the sample to partial adopters and using FEs eliminates household-level unobserved heterogeneity, which provides a robustness check for the effects of RWR2245 adoption on productivity.

Finally, for a subset of outcome variables, i.e. the number of months beans consumed were from own production and purchased in the market, whether the household sells beans, and whether it is a net seller, we evaluate the sensitivity of the findings to different measures of adoption. The alternative measures of adoption are: i) a variable that takes the value of 0, 1, or 2 to reflect the number of seasons in 2015 that a household grew RWR2245, ii) a dummy variable equal to one if a household adopted RWR2245 in both 2015A and 2015B, iii) a count variable for the total number of seasons a household has grown RWR2245, conditional on having grown RWR2245 in 2015, iv) the average percentage of bean land area under RWR2245 between 2015A and 2015B, v) the sum of RWR2245 seeds planted (in kg) in 2015A and 2015B, and vi) a dummy variable equal to one if a household adopted RWR2245 in any season prior to 2015A but did not cultivate RWR2245 in 2015 (this holds true for 9% of households in our sample). For the last estimation, we expect the adoption variable to be either insignificant or have a smaller coefficient than variables measuring adoption in 2015 since adoption in prior seasons should not directly affect bean production in 2015.

## 4 Descriptive statistics

### 4.1 Adoption measures and outcome variables

About 13% of the bush bean varieties planted by bush bean growers in 2015B are RWR2245, 68% are local bush varieties, and 19% are other improved bush varieties, including the other iron-biofortified bush variety, RWR2154, which makes up less than 1% of the observations. About 21% of bush bean growers cultivated RWR2245 in 2015. Distinguishing between seasons reveals that 4% of bush bean growers cultivated RWR2245 in 2015A only, 11% in 2015B only, and 6% in both seasons. Adoption of RWR2245 varies greatly by province; in 2015, 25% of bush bean growing households in the East cultivated RWR2245 compared to 22% in the South, 15% in Kigali, 9% in the West, and 8% in the North.

The average multiplication ratio is 8.25 for RWR2245, 8.26 for other improved bush bean varieties, and 6.74 for local bush bean varieties. The average multiplication ratio for RWR2245 and other improved varieties is significantly higher than that of local varieties. The multiplication ratio for RWR2245 increases with experience growing the variety: the average multiplication ratio for first-time adopters is 7.22 compared to 9.15 for those having grown the variety for more than one season, a difference that is significant at the 10% level. RWR2245 adopters (defined here as households that grew RWR2245 in 2015B) planted 17.18 kg of seeds in 2015B while non-adopters planted 15.84 kg; this difference is not statistically significant, suggesting that adopters do not plant more land to beans than non-adopters.

Households that grew RWR2245 in at least one season in 2015 consumed beans from their production for 8.58 months on average in the past 12 months, which is about one month more than non-adopters did (significant at 5%). RWR2245 adopters purchased beans for about one month fewer than non-adopters (Table 1). However, the average quantity of bean consumed monthly did not vary by adoption status. RWR2245 adopters were more likely to sell beans, sold greater quantities, and were more likely to be net sellers than households that did not grow RWR2245 in 2015. Most households in our sample either sold or purchased beans; only 9.9% were autarkic.

**Table 1:** Descriptive statistics for household-level outcome variables by adoption status in 2015.

	RWR2245 adopters			RWR2245 non-adopters			
Variables	N	Mean	SD	N	Mean	SD	Diff
Qty of bean seed planted in 2015B (kg)	216	17.18	14.91	599	15.84	15.52	
No. of months beans consumed are from own production	243	8.58	3.31	572	7.55	3.33	***
Avg qty of beans consumed monthly when from own production (kg/adult male equivalent)	238	4.07	2.29	566	3.94	2.25	
No. of months beans consumed are from purchases	243	2.78	2.99	572	3.80	3.29	***
Avg qty of beans consumed monthly when from the market (kg/adult male equivalent)	147	3.04	1.81	415	3.34	2.53	
Household sold beans (1 = yes)	243	0.63	0.48	572	0.45	0.50	***
Qty sold, last 12 months (kg)	243	67.91	112.34	572	37.61	87.02	***
Qty sold, last 12 months (kg), *conditional on selling beans*	140	108.37	125.63	244	83.55	114.06	
Net seller (1 = yes)	243	0.55	0.50	572	0.41	0.49	***

*Note:* N refers to the number of observations. SD refers to the standard deviation. Diff refers to the difference in means between adopters and non-adopters and *, **, *** denote the statistical significance at 10%, 5%, and 1% level respectively.

**Fig. 2:**
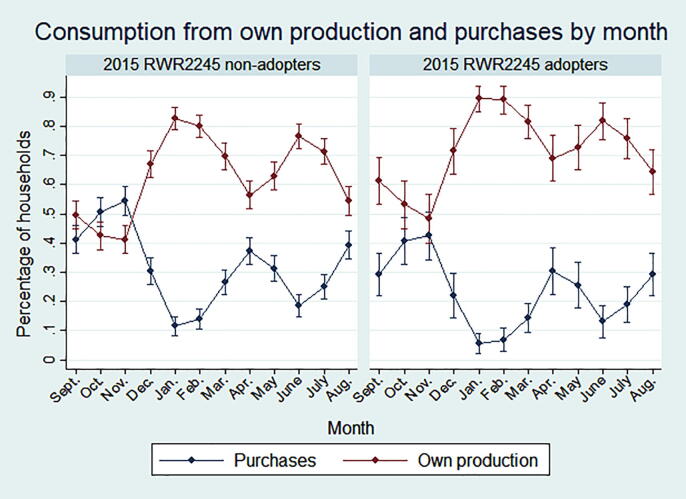
Consumption and purchases of beans by bush bean growers, by month and RWR2245 adoption status.

Fig. 2 shows bean consumption trends by month and adoption status from September 2014 to August 2015. Consumption from own production is most prevalent in January and February, following season A harvest, and in June and July, which follows season B harvest (Asare-Marfo et al., 2016b). From March to May and October to November, which are a few months after seasons A and B harvest, respectively, a greater percentage of adopters consume beans from home production compared to non-adopters.

### 4.2 Explanatory and Instrumental variables

Compared to local bush bean varieties, RWR2245 is less likely to be grown from recycled planting material and more likely to be grown using organic and chemical fertilizer (Table 2). RWR2245 is a recently released variety, which explains why seeds are more frequently purchased compared to varieties that have been cultivated for a longer period of time. Households who cultivated RWR2245 in either season of 2015 were less likely to have grown a climbing bean variety in 2015B than those who did not adopt RWR2245 (Table 3). RWR2245 adopters were also more likely to be literate and live in villages with greater access to extension. The remaining explanatory variables are not statistically different (at the 5% level or lower) between plots under RWR2245 and local bush bean varieties, or between RWR2245 adopters and non-adopters, although the average values of the IVs do vary. Adopters of RWR2245 were more likely to live in a sector with a direct marketing approach, although this difference is significant at the 10% level only (Table 3). Adopters of RWR2245 were also more likely to live in a village where there was a higher proportion of RWR2245 adopters in 2014B and 2015A than non-adopters.

**Table 2:** Descriptive statistics of plot-level variables by variety types in season 2015B.

	RWR2245		Other improved varieties		Local varieties		
Variables	Mean	SD	Mean	SD	Mean	SD	Diff
Recycled seed (1 = yes)	0.27	0.45	0.44	0.48	0.39	0.49	**
Slope							
Flat	0.10	0.30	0.12	0.32	0.08	0.27	
Gentle	0.11	0.32	0.16	0.37	0.14	0.35	
Moderate	0.39	0.49	0.29	0.46	0.35	0.48	
Steep	0.39	0.49	0.43	0.50	0.43	0.49	
Intercrop (1 = yes)	0.60	0.49	0.51	0.50	0.66	0.48	
Walking time to household (minutes)	18.18	33.07	22.20	31.09	15.58	23.07	
Organic fertilizer use (1 = yes)	0.83	0.37	0.71	0.46	0.71	0.45	**
Chemical fertilizer use (1 = yes)	0.17	0.38	0.12	0.33	0.07	0.27	**
Sex of plot decider (1 = female)	0.59	0.49	0.61	0.49	0.61	0.49	
Literacy of plot decider (1 = yes)	0.65	0.48	0.59	0.49	0.59	0.49	
Experience of plot worker (years)	25.92	14.19	26.60	15.40	27.50	16.70	
N	211		222		679		

*Note:* N refers to the number of observations. SD refers to the standard deviation. Diff refers to the difference in means between RWR2245 and local bush bean varieties, and *, **, *** denote the statistical significance at 10%, 5%, and 1% level respectively.

**Table 3:** Descriptive statistics of household-level variables and IVs by RWR2245 adoption status in 2015.

Variables	RWR2245 adopters		RWR2245 non-adopters		
	Mean	SD	Mean	SD	Diff
*Household Characteristics*					
Climbing bean grower (1 = yes)	0.18	0.38	0.27	0.45	***
Distance to city (km)	36.51	21.62	36.09	23.13	
Population density (people/square km)	484.68	537.21	452.54	453.35	
Elevation (10 m)	156.58	15.69	159.16	19.86	
Household size	4.92	2.06	4.87	1.99	
Sex of bean decision maker (1 = female)	0.62	0.49	0.64	0.48	
Literacy of bean decision maker (1 = yes)	0.69	0.46	0.59	0.49	**
Age of bean decision-maker (years)	44.44	13.74	43.84	15.43	
Land size (hectare)	0.54	0.70	0.50	0.75	
Wealth quintiles					
1	0.18	0.39	0.23	0.40	
2	0.16	0.37	0.19	0.41	
3	0.17	0.38	0.20	0.40	
4	0.20	0.41	0.18	0.40	
5	0.28	0.45	0.20	0.39	*
Equipment owned (count)	1.38	0.75	1.21	0.79	*
Livestock (TLU)	0.54	0.88	0.46	1.15	
Access to extension in the village (%)	65.59	24.61	62.06	25.48	**

*Instrumental variables*					
No. of direct marketing in sector 2015A and 2015B	0.47	1.67	0.26	1.05	*
Village RWR2245 adoption rate 2014B (%)	0.11	0.10	0.06	0.08	***
Village RWR2245 adoption rate 2015A (%)	0.18	0.15	0.10	0.10	***
N	243		572		

*Note:* N refers to the number of observations. SD refers to the standard deviation. Diff refers to the difference in means between RWR2245 adopters and non-adopters, and *, **, *** denote the statistical significance at 10%, 5%, and 1% level respectively.

To provide evidence that HarvestPlus did not target areas with greater bean productivity, consumption, or marketing for the dissemination and promotion of iron-biofortified beans either deliberately or by chance, we compare households who live in sectors where direct marketing took place in 2015 with those that do not. If the outcomes of interest and explanatory variables do not differ with household proximity to direct marketing, then this provides evidence that direct marketing is a valid IV. The average multiplication ratio does not differ between households who live in sectors with a direct marketing approach in 2015 and those that do not live in such sectors. Also, none of our higher-order household-level outcome variables vary significantly by whether the household had a direct marketing approach in its sector (Table A1). We also compare plot-level and household-level explanatory variables by households with and without direct marketing in their sector in 2015. Bush bean plots in sectors with direct markets are more likely to have steep slopes and less likely to have moderate or flat slopes than bush bean plots in sectors without direct markets but do not vary by any other plot-level explanatory variables (Table A2). Households in sectors where direct marketing took place live farther from cities (44.11 km vs. 34.80 km) and in villages where a higher percentage of farmers obtain information from extension (77.43% vs. 60.26%) compared to households who did not have direct marketing in their sector in 2015. Other household characteristics, including literacy, wealth, and land under cultivation, do not vary by whether the households had direct marketing in their sector (Table A3). Finally, we compare the proportion of total land in a village that is under bean cultivation between villages in sectors with and without direct marketing. We find that the average share of land devoted to bean cultivation does not vary significantly between the two groups of villages, suggesting that bean cultivation is equally as important in villages in sectors with and without direct marketing (Table A4). The overall similarity between villages in sectors with and without direct markets supports that data on formal delivery approaches can provide a valid IV for RWR2245 adoption.

## 5 Econometrics results

### 5.1 Instrument validity, endogeneity tests, and model fit

Diagnostic tests provide evidence that the IVs are valid (Table A5). The null hypothesis that the model is underidentified is rejected for all regressions based on a Kleibergen-Paap rk LM statistic test. The null hypothesis of weak IVs is rejected for each outcome using the Cragg-Donald F statistic. In all models, the Hansen J test for overidentification fails to reject the null hypothesis that the IVs are valid, indicating that the IVs are not correlated with the error term and correctly excluded from the outcome regressions. The Hausman test fails to reject the null hypothesis that adoption is exogenous in all models, suggesting that adoption is not endogenous to our outcomes of interest.

In the yield regression, the coefficient for the generalized residuals is statistically significant (Table 4), indicating that adoption is endogenous to the multiplication ratio (Wooldridge, 2014).^13^ In all remaining regressions, the p-value of the coefficient for the generalized residuals ranges from 0.356 to 0.892 which is consistent with the Hausman test that suggests that adoption is exogenous. In that case, the non-CFA estimation results are equally valid as the CFA results, and likely more efficient (Wooldridge, 2014). Therefore, for these models, we discuss the results for which adoption is considered exogenous.

**Table 4:** Regression results of the impact of RWR2245 adoption on the multiplication ratio.

	Log of multiplication ratio (quantity harvested/quantity planted)		
	OLS coefficient (robust std. err.)	CFA OLS coefficient (robust std. err.)	FE coefficient (robust std. err.)
Adjusted R2	0.113	0.122	
R2 within			0.145
R2 between			0.002
R2 overall			0.001

Type (base = local)			
Other improved	0.189**	0.207**	0.312**
	(0.081)	(0.080)	(0.137)
RWR2245	0.183**	0.398***	0.182**
	(0.082)	(0.088)	(0.087)
Recycled seed (1 = yes)	0.235***	0.233***	0.191
	(0.062)	(0.061)	(0.121)

Slope (base = steep)			
Moderate	0.138	0.150	0.323
	(0.138)	(0.136)	(0.263)
Gentle	0.044	0.052	0.534**
	(0.130)	(0.129)	(0.266)
Flat	0.145	0.161	0.956***
	(0.128)	(0.127)	(0.226)
Intercrop (1 = yes)	−0.111	−0.096	0.269
	(0.068)	(0.067)	(0.214)
Walk time to household (in minutes)	−0.003*	−0.003*	0.001
	(0.002)	(0.002)	(0.003)
Organic fertilizer use (1 = yes)	−0.097	−0.120	−0.243
	(0.082)	(0.080)	(0.252)
Chemical fertilizer use (1 = yes)	0.087	0.068	0.502**
	(0.113)	(0.113)	(0.236)
Sex (1 = female)	−0.051	−0.059	0.150
	(0.072)	(0.071)	(0.362)
Literate (1 = yes)	0.271***	0.272***	−0.742
	(0.078)	(0.078)	(0.771)
Experience (in years)	0.001	0.001	−0.076
	(0.002)	(0.002)	(0.105)
Elevation (10 m)	−0.001	−0.001	
	(0.002)	(0.002)	
Number of adults	0.004	0.003	
	(0.028)	(0.028)	
Equipment owned (count)	0.107**	0.107**	
	(0.045)	(0.045)	
Extension (%)	0.005***	0.005***	
	(0.002)	(0.002)	
Generalized residual		−0.057***	
		(0.007)	
Constant	0.988*	1.029**	3.168
	(0.386)	(0.381)	(2.199)
N	1112	1112	336

*Note:* * = significance at 10%; ** = significance at 5%; *** = significance at 1%. Standard errors are robust to heteroskedasticity in all models and clustered at the household level for OLS and CFA OLS models. Coefficients for province fixed effects are not shown for brevity.

Based on a likelihood ratio test, the restrictions that the Tobit model holds are rejected (p = 0.000), confirming that the double hurdle model is a better fit than the Tobit model for explaining the quantity sold. The Vuong test also confirms that the zero-inflated Poisson fits the data for the number of months beans were purchased better than the standard Poisson (p = 0.000). After performing PSM, the standardized bias for all covariates is less than 25%, and below 10% for most covariates, which is within recommended limits, indicating that our matching procedures do an adequate job at balancing treatment and control groups and thus controlling for overt bias of the adoption decision (Harder et al., 2010).

### 5.2 Supply indicator results

Holding other factors constant, the multiplication ratio for RWR2245 is 49% (100*(e ^0.398^ –1)) higher than that of local bush varieties according to CFA estimates, and the coefficient is significant at the 1% level (Table 4). In the FE model, the treatment effect of adoption indicates a 20% yield increase over local bush bean varieties, statistically significant at the 5% level. Other improved varieties provide a yield gain of about 23% and 37% over local varieties according to the CFA estimates and FE results, respectively. The PSM indicates that the multiplication ratio for RWR2245 is 23% higher than for local bush bean varieties (Table 6). Being grown from recycled grain, the literacy of the plot decision-maker, ownership of agricultural equipment, and access to extension have a positive effect on the multiplication ratio. In the FE model, which includes fewer observations, growing beans on a flat or gentle slope and applying chemical fertilizer increase the multiplication ratio.

**Table 5:** Regression results of the impact of RWR2245 adoption on the quantity of bean seeds planted in season 2015B.

Log of the quantity of bean seeds planted in 2015B (kg)		
	OLS coefficient (robust std. err.)	CFA OLS coefficient (robust std. err.)
Adj. R2	0.254	0.253
Adopted 2015 (1 = yes)	0.079	0.168
	(0.077)	(0.269)
Climbing bean grower (1 = yes)	0.287***	0.290***
	(0.068)	(0.068)
Distance to city (km)	0.008***	0.008***
	(0.003)	(0.003)
Population density (people/square km)	0.000	0.000
	(0.000)	(0.000)
Elevation (10 m)	−0.009***	−0.009***
	(0.002)	(0.002)
Household size	0.022	0.022
	(0.017)	(0.017)
Sex (1 = female)	−0.101	−0.101
	(0.063)	(0.063)
Literate (1 = yes)	0.044	0.039
	(0.073)	(0.073)
Age (in years)	0.003	0.003
	(0.002)	(0.002)
Land size (ha)	0.232***	0.232***
	(0.086)	(0.086)
Wealth quintile (base = 1)	0.000	0.000
2	0.026	0.022
	(0.096)	(0.096)
3	0.103	0.101
	(0.108)	(0.109)
4	0.330***	0.325***
	(0.117)	(0.117)
5	0.261**	0.254**
	(0.109)	(0.116)
Equipment owned (count)	0.022	0.023
	(0.047)	(0.047)
Livestock (TLU)	0.086***	0.086***
	(0.017)	(0.017)
Extension (%)	−0.004***	−0.004***
	(0.001)	(0.001)
Generalized residual		−0.054
		(0.147)
Constant	3.267***	3.261***
	(0.448)	(0.448)
N	815	815

*Note:* * = significance at 10%; ** = significance at 5%; *** = significance at 1%. Standard errors are robust to heteroskedasticity and clustered at the village level. Coefficients for province fixed effects are not shown for brevity.

**Table 6:** Matching results for the impact of RWR2245 adoption on the multiplication ratio and quantity of bean seeds planted in season 2015B.

Matching algorithm	Multiplication ratio	Bean seeds planted (kg)
Radius	0.232 (0.075) ***	0.074 (0.068)
Kernel	0.228 (0.069) ***	0.083 (0.075)
N	890	815

*Note:* Standard errors are bootstrapped with 200 replications. Caliper is set to 0.01 for all matching algorithms.

The average multiplication ratio for local varieties is 6.74 and on average, bush bean growers planted 15.57 kg of bush bean seed in 2015B; if this seed were entirely local bush varieties, the average household would harvest 104.94 kg of beans. Converting all planted bush bean seed from local bush varieties to RWR2245 would provide a harvest of 156.36 kg, based on a 49% yield gain from RWR2245 over local bean bush varieties (estimate obtained from the CFA). This corresponds to an additional 51.42 kg of beans per season compared to no adoption of improved varieties. When excluding RWR2245, 78% of bush bean varieties grown in 2015B were local varieties and 22% were other improved varieties. Taking this into account, and assuming no difference in multiplication ratio between RWR2245 and other improved varieties, the average additional harvest from RWR2245 adoption is about 38%; this corresponds to an additional 40.11 kg per season or about 80 kg per year. According to our community survey data, the average price of beans in the sampled villages is 337.58 RWF, meaning that the additional 80 kg has a market value of about 27,000 RWF, which is equivalent to US$28.81. Given that the GDP per capita in Rwanda in 2015 was $728.10 (The World Bank, 2019), this potential income gain (from sales or freeing income due to lower bean purchases) represents 3.8% of the GDP per capita. Unfortunately, our data do not provide detailed information on production costs, and thus, we cannot estimate the net increase in income from RWR2245 adoption. The average cost of RWR2245 seed (381.49 RWF) is very similar to that of local varieties (351.53 RWF) and other improved varieties (366.49 RWF). However, other input costs could vary, particularly since the descriptive statistics show that farmers are more likely to apply fertilizer to plots planted with RWR2245 compared to those planted to local bush bean varieties.

Adoption of RWR2245 has no effect on the quantity of bean seed planted (Table 5, Table 6), suggesting that adoption does not lead to changes in the proportion of land devoted to bean cultivation. Adopters, therefore, obtain higher quantities of harvested grain as a result of adoption. This extra production can lead to higher-order outcomes such as greater quantity consumed and/or sold, likely improving household nutrition. Households that live farther from cities, have more household members, cultivate more land, own more livestock, and are wealthier have more land under bean cultivation while households at higher elevations have less land devoted to beans.

### 5.3 Consumption and market indicator results

RWR2245 adoption (season 2015A, B or both) increases bean consumption from home production by 0.64 months (equivalent to 19–20 days) (Table 7) while reducing the length of time beans have to be purchased by 0.73 months (equivalent to 22–23 days) in a year (Table 8). However, adoption of RWR2245 has no effect on the average quantity consumed monthly when beans are sourced from own production or the market (Table 7, Table 8). This suggests that adoption does not change the quantity consumed, but changes the source of beans consumed, moving away from purchases towards own production. This result on its own is important since it suggests that the adoption of RWR2245 leads to an increase in iron intake among adopting households, as RWR2245 is higher in iron than other varieties that households can purchase in the market. As an additional robustness check, we estimated a regression with the log of the sum of beans consumed from own production and purchases as the dependent variable. We find no effect of RWR2245 adoption on the total quantity of beans consumed in the last 12 months.

**Table 7:** Regression results of the impact of RWR2245 adoption on the number of months households consume beans from own production and average quantity consumed monthly from own production.

	Months consumed from own production		Quantity consumed monthly (kg per adult equivalent)	
	Poisson marginal effects (Delta method std. err.)	CFA Poisson marginal effects (Delta method std. err.)	OLS coefficient (robust std. err.)	CFA OLS coefficient (robust std. err.)
Adj. R2			0.107	0.107
Log pseudolikelihood	−2427222.7	−2426875.1		
Adopted 2015 (1 = yes)	0.641**	1.392	0.012	0.079
	(0.287)	(1.234)	(0.057)	(0.235)
Climbing bean grower (1 = yes)	0.752**	0.795***	−0.033	−0.029
	(0.291)	(0.294)	(0.054)	(0.054)
Distance to city (km)	0.026***	0.027***	0.000	0.000
	(0.009)	(0.011)	(0.002)	(0.002)
Population density (people/square km)	−0.001***	−0.001***	0.000	0.000
	(0.000)	(0.000)	(0.000)	(0.000)
Elevation (10 m)	−0.021***	−0.020***	−0.001	−0.001
	(0.007)	(0.007)	(0.001)	(0.001)
Household size	−0.144**	−0.144**	−0.094***	−0.094***
	(0.070)	(0.071)	(0.011)	(0.011)
Sex (1 = female)	−0.368	−0.376	−0.064	−0.065
	(0.272)	(0.268)	(0.049)	(0.048)
Literate (1 = yes)	0.571*	0.518*	0.178***	0.174***
	(0.303)	(0.299)	(0.059)	(0.063)
Age (years)	0.026***	0.032***	0.001	0.001
	(0.007)	(0.010)	(0.001)	(0.001)
Land size (ha)	0.613***	0.616***	0.046	0.046
	(0.125)	(0.124)	(0.032)	(0.032)
Wealth quintile (base = 1)				
2	0.140	0.128	0.062	0.061
	(0.450)	(0.453)	(0.079)	(0.079)
3	0.237	0.236	0.020	0.020
	(0.469)	(0.470)	(0.077)	(0.077)
4	0.947**	0.913**	−0.024	−0.027
	(0.434)	(0.442)	(0.068)	(0.069)
5	1.761***	1.701***	−0.171**	−0.176**
	(0.473)	(0.496)	(0.077)	(0.075)
Equipment owned (count)	0.636***	0.592***	0.027	0.024
	(0.138)	(0.156)	(0.025)	(0.032)
Livestock (TLU)	0.067	0.075	0.069***	0.069***
	(0.077)	(0.078)	(0.012)	(0.013)
Extension (%)	0.005	0.004	−0.001	−0.001
	(0.005)	(0.005)	(0.001)	(0.001)
Generalized residual		−0.458		−0.040
		(0.719)		(0.144)
Constant (coefficient)	2.028***	2.020***	1.736***	1.731***
	(0.182)	(0.183)	(0.227)	(0.233)
N	815	815	804^a^	804

*Note:* * = significance at 10%; ** = significance at 5%; *** = significance at 1%. Standard errors are robust to heteroskedasticity and clustered at the village level. Coefficients for province fixed effects are not shown for brevity.

a Eleven households did not consume beans from own production in any months.

**Table 8:** Regression results of the impact of RWR2245 adoption on the number of months households consume beans from purchases and the average quantity purchased monthly.

	Months purchased		Quantity purchased monthly (kg per adult equivalent)	
	Zero-inflated Poisson marginal effects (Delta method std. err.)	CFA Zero-inflated Poisson marginal effects (Delta method std. err.)	OLS coefficient (robust std. err.)	CFA OLS coefficient (robust std. err.)
Adj. R2			0.175	0.174
Log pseudolikelihood	−1995457	−1994473		
Adopted 2015 (1 = yes)	−0.728***	−0.876	−0.102	−0.171
	(0.263)	(1.141)	(0.071)	(0.305)
Climbing bean grower (1 = yes)	−0.270	−0.271	−0.060	−0.063
	(0.275)	(0.280)	(0.067)	(0.069)
Distance to city (km)	−0.019**	−0.019*	0.001	0.001
	(0.009)	(0.010)	(0.002)	(0.002)
Population density (people/square km)	0.001***	0.001***	−0.000	−0.000
	(0.000)	(0.000)	(0.000)	(0.000)
Elevation (10 m)	0.013**	0.013**	−0.000	−0.000
	(0.006)	(0.006)	(0.002)	(0.002)
Household size	0.187***	0.187***	−0.103***	−0.103***
	(0.055)	(0.055)	(0.015)	(0.015)
Sex (1 = female)	0.142	0.139	−0.010	−0.010
	(0.276)	(0.276)	(0.064)	(0.064)
Literate (1 = yes)	−0.492**	−0.482**	0.177**	0.181**
	(0.229)	(0.238)	(0.075)	(0.074)
Age (years)	−0.031***	−0.031***	0.003	0.003
	(0.008)	(0.008)	(0.002)	(0.002)
Land size (ha)	−0.460***	−0.457***	0.068	0.068
	(0.172)	(0.172)	(0.044)	(0.044)
Wealth quintile (base = 1)				
2	0.189	0.197	0.106	0.107
	(0.365)	(0.365)	(0.086)	(0.085)
3	−0.025	−0.031	0.078	0.078
	(0.398)	(0.397)	(0.098)	(0.098)
4	−0.630*	−0.630*	−0.060	−0.057
	(0.368)	(0.372)	(0.091)	(0.094)
5	−1.359***	−1.374***	−0.006	−0.001
	(0.395)	(0.423)	(0.094)	(0.095)
Equipment owned (count)	−0.517***	−0.511***	0.125***	0.128**
	(0.175)	(0.185)	(0.045)	(0.049)
Livestock (TLU)	−0.893***	−0.889***	−0.075	−0.074
	(0.284)	(0.285)	(0.075)	(0.074)
Extension (%)	0.006	0.006	−0.003*	−0.003*
	(0.005)	(0.005)	(0.002)	(0.002)
Generalized residual		0.085		0.042
		(0.664)		(0.177)
Constant	1.861***	1.795***	1.283***	1.291***
	(0.973)	(0.969)	(0.332)	(0.335)
N	815	815	562	562

*Note:* * = significance at 10%; ** = significance at 5%; *** = significance at 1%. Standard errors are robust to heteroskedasticity and clustered at the village level. Coefficients for province fixed effects are not shown for brevity.

Adopting RWR2245 increases the probability of selling beans by 12% and increases the quantity of beans sold by 17.28 kg, although the effect on quantity sold is significant at the 10% level only (Table 9). Given that households sell on average 44 kg of beans per year, this represents an increase in sales of about 38%. RWR2245 adopters are also 8% more likely to be net sellers of beans, with the coefficient being significant at the 10% level. The low levels or lack of significance of adoption on quantities consumed, purchased, and sold could be partially due to difficulties for the respondents to recall and correctly estimate these quantities over 12 months. However, it should be easier to remember when the household consumed beans from own production and purchases and whether they sold beans. This may also affect the precision and magnitude of our estimate of the impact of adoption on the likelihood of being a net seller of beans. It is important to note that in the model specifications for the consumption and market indicators which include the generalized residuals, RWR2245 adoption is not significant. Therefore, our significant results hold only in our preferred specifications which do not include the generalized residuals.

**Table 9:** Regression results of the impact of RWR2245 adoption on the probability of selling beans, quantity sold, and the probability of being a net seller.

	Sold beans (1 = yes)		Quantity sold (kg)		Net seller (1 = yes)	
	Probit marginal effects (Delta method std. err.)	CFA Probit marginal effects (Delta method std. err.)	Double hurdle average partial effects (bootstrapped std. err.)	CFA Double hurdle average partial effects (bootstrapped std. err.)	Probit marginal effects (Delta method std. err.)	CFA Probit marginal effects (Delta method std. err.)
Pseudo R2	0.147	0.147			0.192	0.193
Log psuedolikelihood	−562430.16	−562421.25	−3047935.5	−3047091.6	−852005.65	−526694.95
Adopted 2015 (1 = yes)	0.122***	0.103	17.276*	49.202	0.081*	0.005
	(0.047)	(0.145)	(9.081)	(40.400)	(0.047)	(0.139)
Climbing bean grower (1 = yes)	0.024	0.023	−2.991	−1.062	0.068	0.064
	(0.044)	(0.045)	(9.503)	(10.872)	(0.043)	(0.044)
Distance to city (km)	0.004***	0.004***	1.284***	1.324***	0.005***	0.005***
	(0.001)	(0.001)	(0.339)	(0.334)	(0.001)	(0.001)
Population density (people/square km)	−0.000*	−0.000*	−0.006	−0.004	−0.000**	−0.000**
	(0.000)	(0.000)	(0.011)	(0.011)	(0.000)	(0.000)
Elevation (10 m)	−0.005***	−0.005***	−0.732***	−0.672***	−0.003***	−0.003***
	(0.001)	(0.001)	(0.268)	(0.258)	(0.001)	(0.001)
Household size	−0.004	−0.004	0.184	−0.131	−0.031***	−0.031***
	(0.010)	(0.010)	(2.030)	(2.084)	(0.010)	(0.010)
Sex (1 = female)	−0.043	−0.042	−17.436*	−18.132*	−0.017	−0.016
	(0.042)	(0.042)	(9.129)	(10.741)	(0.043)	(0.043)
Literate (1 = yes)	0.022	0.024	20.276*	17.425*	0.005	0.010
	(0.043)	(0.044)	(10.476)	(9.169)	(0.044)	(0.043)
Age (years)	−0.002	−0.002	−0.287	−0.316	0.002*	0.002*
	(0.001)	(0.001)	(0.296)	(0.288)	(0.001)	(0.001)
Land size (ha)	0.043	0.043	14.609**	14.475***	0.070**	0.070*
	(0.033)	(0.033)	(6.215)	(5.313)	(0.036)	(0.036)
Wealth quintile (base = 1)						
2	0.013	0.014	−16.766	−16.698	−0.031	−0.030
	(0.053)	(0.053)	(23.452)	(22.319)	(0.052)	(0.052)
3	−0.006	−0.006	−0.239	−0.550	0.014	0.014
	(0.064)	(0.064)	(13.040)	(13.798)	(0.055)	(0.054)
4	0.099	0.100	10.911	8.829	0.115**	0.118**
	(0.068)	(0.068)	(15.038)	(15.189)	(0.055)	(0.056)
5	0.109	0.110	27.996	25.433	0.254***	0.260***
	(0.072)	(0.072)	(17.186)	(16.682)	(0.064)	(0.067)
Equipment owned (count)	−0.022	−0.021	7.881	5.415	0.062**	0.066**
	(0.027)	(0.029)	(5.437)	(5.943)	(0.030)	(0.030)
Livestock (TLU)	−0.033	−0.033	−0.634	−0.528	0.118***	0.119***
	(0.028)	(0.028)	(5.066)	(6.569)	(0.042)	(0.042)
Extension (%)	0.001**	0.001**	0.076	0.029	0.000	0.000
	(0.000)	(0.000)	(0.161)	(0.169)	(0.000)	(0.001)
Generalized residual		0.012		−19.754		0.046
		(0.086)		(19.017)		(0.077)
Constant (coefficient)	2.120***	2.126***	−1319.285	−1521.003	0.429	0.453
	(0.616)	(0.608)	(1433.048)	(1522.696)	(0.540)	(0.534)
N	815	815	815	815	815	815

*Note:* * = significance at 10%; ** = significance at 5%; *** = significance at 1%. Standard errors are robust to heteroskedasticity and clustered at the village level. Coefficients for province fixed effects are not shown for brevity.

Climbing bean growers consume beans from their production for more months than those that grow bush beans only, which is not surprising given the yield advantage of climbers over bush beans and is consistent with previous findings (Katungi et al., 2018). Distance from an urban area has a positive effect on the number of months beans are consumed from own production, selling and being a net seller, and a negative effect on the number of months beans are purchased. Population density and elevation have a negative effect on the number of months beans are consumed from own production but a positive effect on the number of months a household purchases beans. Households at higher elevation are also less likely to sell beans or be net sellers of beans. Household size has a positive effect on the number of months beans are purchased, but a negative effect on the average quantity purchased per month, the number of months beans are consumed from own production, and being a net seller of beans. Literacy of the respondent has a negative effect on the number of months beans are purchased but a positive influence on the average monthly quantity consumed from purchases and own production. The age of the respondent increases the number of months beans are consumed from their production while reducing the number of months beans are purchased. Households that cultivate more land consume beans from their production for a greater number of months, purchase beans for fewer months, sell higher quantities of beans, and are more likely to be net sellers of beans. The wealth index and the number of pieces of agricultural equipment owned have a positive effect on the number of months households consume beans from their production and a negative effect on the number of months beans are purchased. The wealth index also has a negative impact on the average monthly quantity consumed from own production while agricultural equipment ownership has a positive effect on the average monthly quantity purchased. Livestock ownership has a positive effect on the average monthly quantity consumed from own production but a negative influence on the number of months a household purchases beans. Finally, access to agricultural extension makes households more likely to sell beans.

The results of PSM analysis are consistent with the previous findings, providing evidence of the robustness of our results to econometric methods (Table 10). According to PSM results, adoption of RWR2245 increases the number of months households consume beans from own production by 0.62–0.64 months, reduces the number of months households purchase beans by 0.66–0.69 months, increases the likelihood of selling beans by 12–13% and the probability of being a net seller of beans by 9–10% and finally, increases bean sales by an additional 18–21 kg per year, or 41–48% over the average quantity sold.

**Table 10:** Matching results for the impact of RWR2245 adoption on bean consumption from own production, purchases, and marketing.

Matching algorithm	Months consumed from own production	Quantity consumed monthly (kg per adult equivalent)	Months purchased	Quantity purchased monthly (kg per adult equivalent)	Sold beans (1 = yes)	Quantity sold (kg)	Net seller (1 = yes)
Radius	0.621 (0.269)**	0.029 (0.047)	−0.662 (0.243)***	−0.051 (0.068)	0.120 (0.042)***	18.409 (8.834)**	0.093 (0.039)**
Kernel	0.644 (0.249)**	0.032 (0.046)	−0.686 (0.229)***	−0.061 (0.057)	0.129 (0.040)***	20.675 (8.102)**	0.101 (0.037)***
N	815	804	815	562	815	815	815

*Note:* Standard errors are bootstrapped with 200 replications. Caliper is set to 0.01 for all matching algorithms, and only observations that fall under common support are included.

Alternative specifications of adoption also provide consistent results, providing further indication that our results are highly robust not only to different estimation methods but also to different measures of adoption (Table 11). If adoption is defined as a count variable equal to the number of seasons in 2015 that a household grew RWR2245 (0, 1, or 2), results of the Poisson, zero-inflated Poisson, and probit regressions, respectively, indicate that growing RWR2245 for an additional season increases consumption from own production by 0.55 months, reduces the number of months beans are purchased by 0.64 months, and increases the probability of selling beans and being a net seller by 11% and 8%, respectively (each significant at the 5% level or lower). If we consider as adopters only households that grew RWR2245 in both seasons of 2015, adoption increases beans consumption from own production by 1.21 months, reduces bean purchases by 1.39 months, and increases the probability of selling beans and being a net seller by 19% and 21%, respectively, roughly doubling the estimated effects of growing RWR2245 for one season. Increasing the proportion of bean land under RWR2245 by 10% increases (decreases) bean consumption from own production (purchases) by 0.02 months (about half a day) and increases the probability of selling beans and being a net seller of beans by 0.2%. Planting an additional kg of RWR2245 seeds in 2015A or 2015B increases consumption from own production by 0.02 months and increases the probability of selling beans and being a net seller by 1% each. Conditional on having planted RWR2245 in 2015A or 2015B, growing RWR2245 an additional season prior to 2015 increases bean consumption from own production by 0.18 months, reduces the need to purchase beans by 0.26 months, and increases the probability of being a net seller by 3%, meaning that the benefits of RWR2245 adoption increase as households gain more experience growing the variety. Finally, having grown RWR2245 prior to 2015A, but not being an adopter in 2015, does not have a significant impact on the number months beans are consumed from own production and purchases or on the probability of being a net seller of beans, although it does increase the likelihood of selling beans by 12%. Thus, regardless of how adoption is measured, results indicate that adopters replace bean purchases with beans from own production that are higher in iron and generate a surplus to sell, potentially improving nutrition via a consumption and income pathway.

**Table 11:** Alternative measures of adoption and their impact on bean consumption from own production, purchases, and marketing.

	Months of consumption from own production	Months of purchasing	Sell beans (1 = yes)	Net seller of beans (1 = yes)
	Poisson marginal effects (Delta method std. err.)	ZIP Poisson marginal effects (Delta method std. err.)	Probit marginal effects (Delta method std. err.)	Probit marginal effects (Delta method std. err.)
Adoption in both seasons	1.207***	−1.387***	0.182***	0.210***
	(0.425)	(0.499)	(0.063)	(0.057)
Adoption in 0,1, or 2 seasons of 2015	0.552***	−0.642***	0.096***	0.080***
	(0.202)	(0.202)	(0.031)	(0.030)
Number of seasons growing RWR2245 (conditional on growing RWR2245 in 2015A or 2015B)	0.187***	−0.265**	0.018	0.029**
	(0.078)	(0.108)	(0.015)	(0.013)
% Bean area under RWR2245, average between 2015A and 2015B	0.016**	−0.018***	0.002**	0.002**
	(0.006)	(0.007)	(0.001)	(0.001)
Quantity of RWR2245 planted (sum of 2015A and 2015B, in kg)	0.023**	−0.024	0.011**	0.010**
	(0.011)	(0.029)	(0.003)	(0.003)
Household grew RWR2245 in a season prior to 2015A	0.448	−0.326	0.121**	0.090*
	(0.324)	(0.331)	(0.050)	(0.053)

*Note:* * = significance at 10%; ** = significance at 5%; *** = significance at 1%. Standard errors are robust to heteroskedasticity and clustered at the village level.

RWR2245 adoption increases bean consumption from own production by 1.21 months and household monthly bean consumption averages 12.99 kg when beans consumed are from own production. This requires an additional 15.98 kg of beans harvested over seasons 2015A and 2015B. This is well within reach given the estimated yield gains of 80 kg over two seasons from converting local bush bean varieties to RWR2245, and adopters would also have a surplus to sell. Therefore, adoption should improve iron intake and household nutrition through the substitution in consumption of non-biofortified bean varieties with RWR2245. By reducing quantity purchased for net buyers and increasing quantity sold for net sellers of beans, adoption can also improve nutrition by increasing income to spend on other foods and health-related goods for both types of households. Our data shows that households consume on average 66% of their bean production and this average does not vary whether the beans are local, RWR2245, or other improved varieties. Thus, the larger share of RWR2245 production is consumed by adopting households. Approximately 3% and 14% of bean production is given away and sold, respectively (these figures do not vary by bean variety types).^14^ Thus, a non-negligible portion of RWR2245 grains reaches the market, increasing the iron content of beans in the market and leading to nutritional spillover effects among households that purchase beans.

## 6 Conclusions

Biofortified crops are a relatively new and expanding technology developed to address the global challenge of micronutrient deficiency. It is thus important to understand the impact this technology has on households who produce and consume these crops, especially considering the complex pathways through which agricultural interventions can affect household nutrition and well-being. This paper provides highly robust evidence that biofortified crops can improve household nutrition via two main pathways. The first is by increasing nutrient intake via increased consumption of the own produced biofortified crop, which is higher in iron than other bean varieties available to the household to grow or purchase in the market. The second pathway occurs by increasing household income available for the purchase of other nutritious foods via reductions in purchases and increased sales of the targeted crop. These two pathways are made possible by the enhanced nutritive value of the biofortified crops and their high-yielding properties which boost harvested quantity.

More specifically, we find that adoption of the most popular iron-biofortified bean variety, RWR2245, increases yields by 20–49% over traditional bush bean varieties in Rwanda; this range of estimates from different estimation methods likely provides a lower and upper bound of the yield effect. This productivity advantage is crucial for sustained adoption of RWR2245 and a critical determinant of overall impacts. Having rigorously quantified the productivity gain strengthens the attribution of the higher-order impacts to the adoption of RWR2245. Households who grow RWR2245 in one or two seasons consume beans from their production for an additional 0.62–0.64 months (about 19–20 days) per year compared to those that did not grow RWR2245. RWR2245 adoption also reduces the need to purchase beans by 0.66–0.92 months (about 20–28 days) over 12 months. Adoption allows households to shift the source of bean consumption away from purchases toward own production, suggesting that the biofortified variety leads to an increase in iron intake among adopters. In addition, adoption increases the likelihood that a household sells beans by 12–14%, which increases the availability of iron-rich foods in the markets, also benefiting those who purchase beans. These results are robust to a variety of estimation methods and specifications for the adoption decision, which provides a high level of confidence in our findings.

Women aged 19–50 need to consume about 18 mg of iron per day (Institute of Medicine, 2018). For an average household, switching all beans consumed from own production from non-biofortified to biofortified would lead to a substantial increase in iron intake. Glahn et al. (2017) estimate that one kg of non-biofortified beans contains 47.5 mg/kg of iron while iron-biofortified beans contain 82.5 mg/kg. Adopting households consume on average 4.07 kg of beans per adult equivalent per month when beans are sourced from own production, which is equivalent to 135 g/day. This consumption level would provide 6.42 mg of iron per adult equivalent per day if beans consumed are non-biofortified compared to 11.15 mg for biofortified beans. This additional 4.7 mg of iron would bring women much closer to their daily requirement of 18 mg per day, lowering their risk of iron deficiency. Iron deficiency can result in reduced cognitive performance and worker productivity, increased maternal mortality, and impaired immune systems, among other consequences, and improving iron status has been found to reverse many of these negative effects (World Health Organization, 2001).

Policymakers and researchers can use these results to inform the promotion and targeting of biofortified crop delivery in Rwanda and elsewhere. By increasing consumption from own production, the adoption of iron-biofortified beans directly boosts nutrient intakes among adopting households. This means that targeting households with high nutritional needs during dissemination efforts would further increase the health benefits associated with biofortification. Adoption also increases income available to spend on other foods regardless of whether the household is a net purchaser or net seller of beans. Surplus sold in the marketplace, which is made possible due to the high-yielding property of the iron-biofortified variety, leads to nutritional spillover benefits, indicating that biofortified crops can enhance nutrition through several channels.

Our paper provides evidence that biofortification is a promising intervention for improving the nutrition and health of rural populations and supports the need for further research to quantify the impacts of adoption on food consumption patterns and nutrient intake. In countries such as Rwanda where households have both high consumption and production of the targeted crop, biofortification has the potential to provide substantial benefits for both adopting households and their surrounding communities.

## Credit authorship contribution statement

**Kate Vaiknoras:** Conceptualization, Methodology, Software, Validation, Formal analysis, Investigation, Data curation, Writing - original draft, Writing - review & editing, Visualization. **Catherine Larochelle:** Conceptualization, Methodology, Software, Validation, Formal analysis, Writing - review & editing, Supervision, Project administration, Funding acquisition.

## Declaration of Competing Interest

The authors declare that they have no known competing financial interests or personal relationships that could have appeared to influence the work reported in this paper.

## Footnotes

1 If there were fewer than six iron-biofortified bean adopters in a village, additional non-adopters were interviewed to reach a total of 12 households interviewed in the village.

2 Potential bias could arise if some of the bush bean growers in our sample lived in areas that mainly grew climbing beans: RWR2245 adoption could be lower in these areas while at the same time less well adapted to these areas. To investigate this, we identify villages where bush beans are not commonly grown (villages where fewer than half of sampled households cultivated bush beans in 2015B). We re-estimated each of our models with these villages dropped and found that this does not substantially change our results. We therefore conclude that differences in areas where bush beans are well adapted vs. those that are not do not bias our main results.

3 This is the only season for which we have complete information on all bean varieties the households grew.

4 Three variety observations were dropped from the analysis because households reported that they were RWR2245 but responses to other questions cast doubt on this. For instance, households reported first growing RWR2245 before its release and reported the wrong seed color. In addition, we excluded three households from the analyses because of outlier values for the outcome variables. We re-estimated the models including these dropped observations and our results and conclusions did not change.

5 The income effect will be attenuated if RWR2245 has higher production costs compared to local varieties, which we discuss later on in the section on econometric results.

6 One could argue that autarkic households after adoption may become net buyers if their income elasticity of demand for beans is greater than 1 and they do realize an increase of their income through higher yields. However, given that per capita bean consumption is very high in Rwanda, the income elasticity of demand for bean is likely to be less than one.

7 Planting density is similar between different bush bean varieties.

8 Adult male equivalents are calculated based on a scale obtained by USAID Dary, O., Hainsworth, M., 2008. The Food Fortification Formulator: Technical Determination of Fortification Levels and Standards for Mass Fortification. USAID, Washington, DC.

9 This includes asset ownership (motorcycle/scooter, bicycle, cell phone, radio, and television) and housing characteristics (glass windows, access to electricity, outer wall material) and source of drinking water.

10 1 cow = 0.5; 1 sheep = 0.1; 1 goat = 0.1; 1 pig = 0.2; 1 chicken = 0.01; 1 rabbit = 0.02.

11 Baum (2013) notes that a limitation of CFA is that when both the outcome variable and endogenous regressor are binary, the first stage model must be correctly specified or the estimator may be inconsistent. This applies to our models that examine the probability of selling beans and the probability of being a net seller of beans; these models therefore require that the first stage is correctly specified.

12 We use the user-written Stata commands psmatch2 and pstest to perform PSM and assess post-balance matching, respectively Leuven and Sianesi (2003). PSMATCH2: Stata module to perform full Mahalanobis and propensity score matching, common support graphing, and covariate imbalance testing.

13 Wooldridge (2015) states that if the coefficient of the generalized residual is statistically significant, the standard errors should be adjusted or bootstrapped. Due to the difficulty of bootstrapping standard errors while using sampling weights, we did not bootstrap our standard errors for the multiplication ratio regression, as sampling weights are crucial for our analysis. However, we did estimate this model without sampling weights both with and without bootstrapped errors. We found that bootstrapping made only minimal changes to our standard errors. We therefore conclude that bootstrapping would not change our main conclusions.

14 Fourteen percent of harvest is saved as seed for the next growing season. The remaining three percent represents other usage or loss during storage.
